# Anatase TiO_2_ ultrathin nanobelts derived from room-temperature-synthesized titanates for fast and safe lithium storage

**DOI:** 10.1038/srep11804

**Published:** 2015-07-02

**Authors:** Wei Wen, Jin-ming Wu, Yin-zhu Jiang, Sheng-lan Yu, Jun-qiang Bai, Min-hua Cao, Jie Cui

**Affiliations:** 1 State Key Laboratory of Silicon Materials, Key Laboratory of Advanced Materials and Applications for Batteries of Zhejiang Province, and School of Materials Science and Engineering, Zhejiang University, Hangzhou 310027, P. R. China; 2 Key Laboratory of Cluster Science, Ministry of Education of China, and Department of Chemistry, Beijing Institute of Technology, Beijing 100081, P. R. China; 3 College of Mechanical and Electrical Engineering, Hainan University, Haikou 570228, P. R. China; 4 Department of Physics, Wenzhou University, Wenzhou 325035, P. R. China

## Abstract

Lithium-ion batteries (LIBs) are promising energy storage devices for portable electronics, electric vehicles, and power-grid applications. It is highly desirable yet challenging to develop a simple and scalable method for constructions of sustainable materials for fast and safe LIBs. Herein, we exploit a novel and scalable route to synthesize ultrathin nanobelts of anatase TiO_2_, which is resource abundant and is eligible for safe anodes in LIBs. The achieved ultrathin nanobelts demonstrate outstanding performances for lithium storage because of the unique nanoarchitecture and appropriate composition. Unlike conventional alkali-hydrothermal approaches to hydrogen titanates, the present room temperature alkaline-free wet chemistry strategy guarantees the ultrathin thickness for the resultant titanate nanobelts. The anatase TiO_2_ ultrathin nanobelts were achieved simply by a subsequent calcination in air. The synthesis route is convenient for metal decoration and also for fabricating thin films of one/three dimensional arrays on various substrates at low temperatures, in absence of any seed layers.

The drastic depletion of traditional fossil energies and serious environmental pollutions stimulate tremendous research interests nowadays to develop sustainable energies and advanced energy-storage systems. Among various energy-storage systems, lithium-ion batteries (LIBs) are promising candidates for portable electronics, future electric vehicles/hybrid electric vehicles, and power-grid applications because of its high energy density^1^,^2^,^3^. The state-of-the-art anode material for LIBs, graphite, cannot fulfill the demands for large scale energy storage applications due to its poor rate performance and serious safety issues. Moreover, abundance, toxicity, synthetic methods and scalability of anode material should be considered to reduce the energy and environmental costs of LIBs^3^. Significant efforts have been made on Ti-based compounds, for example Li_4_Ti_5_O_12_ and TiO_2_, as anodes for LIBs because of their **superior safety** as well as resource abundance and non-toxicity^4^,^5^,^6^. The inherent overcharge protection of Ti-based compounds arises from the fact that high Li-intercalation potential avoids the lithium dendrites and formation of the solid electrolyte inerphase (SEI) film as well. Also served as important functional material in photocatalysis^7^, energy conversion^8^, and gas sensors^9^, TiO_2_ possesses a theoretical capacity of 335 mAhg^−1^, which is much higher than that of Li_4_Ti_5_O_12_ (175 mAhg^−1^) having been successfully commercialized. Full cells constructed with TiO_2_ and LiFePO_4_ or LiNi_0.5_Mn_1.5_O_4_ were reported to exhibit fascinating performances^10^. Among common TiO_2_ polymorphs of anatase, rutile, and brookite, anatase presents the most excellent properties in lithium storage^11^ and photocatalysis^12^. However, for lithium storage, anatase TiO_2_ still suffers from low capacity and poor rate performance because of the sluggish Li ion diffusion and poor electronic conductivity^11^.

Reducing the dimension to a nanometer-scale range is one of the most effective strategies for anatase to improve the battery performance^13^,^14^,^15^,^16^,^17^,^18^,^19^, because the characteristic time constant *t* for diffusion is proportional to the square of the diffusion length *L* (*t *≈* L*^*2*^*D*^−*1*^)^20^. Specifically, two-dimensional (2D) nanomaterials can provide greatly increased electrolyte/electrode contact area and effectively shorten diffusion distance of Li ions, which in turn enhances their electrochemical performances^21^,^22^,^23^. For example, anatase TiO_2_ nanosheets with exposed highly reactive (001) facets exhibited excellent properties for lithium storage^24^. Ultrathin 2D nanomaterials demonstrate many unique physical and chemical properties^25^. As a special nanostructure, nanobelts integrate the merits of 2D nanomaterials with enhanced charge transport that is characteristic of one-dimensional (1D) configuration.

Anatase TiO_2_ nanobelts are typically fabricated via calcinations of hydrogen titanate, which is prepared by an alkaline hydrothermal treatment followed by a subsequent proton exchange^26^,^27^,^28^,^29^. Utilizing titanate derived by such a multi-step procedure as intermediates, TiO_2_ with other 1D and 2D nanostructures could be obtained^30^,^31^,^32^,^33^,^34^,^35^,^36^. Recently, a vapour-phase hydrothermal method using ammonia instead of NaOH was developed for the direct growth of titanate nanotubes on a titanium substrate^37^. Mechanical agitation was also introduced into the alkaline hydrothermal technique to obtain elongated titanate nanotubes^38^. However, there are potential dangers in a high-pressure route, especially in large-scale productions, and even unfortunately, the anatase TiO_2_ nanobelts by alkaline hydrothermal reactions usually exhibit a large thickness and low surface area^26^,^27^, which is not favored for the lithium storage. Certain low temperature techniques have been developed to synthesize titanate, including those with a nanosheet structure^39^; unfortunately, the sheet is still **not thin enough** and the specific surface area is also not high. It is still challenging to obtain TiO_2_ with high performance for lithium storage via a simple and scalable synthesis method.

Herein, we present the synthesis of hydrogen titanate ultrathin nanobelts via a novel and robust H_2_O_2_-asisisted wet-chemistry route at ambient conditions, as illustrated schematically in Fig. 1. An amorphous black Ti-based precursor was prepared via a rapid pyrolysis process, which, unlike common Ti-based precursor, is quite **water-proof** and hence easy to handle. Simply immersing the precursor in aqueous H_2_O_2_ under the ambient conditions resulted in the formation of hydrogen titanate ultrathin nanobelts. With the morphology remained stable, the hydrogen titanate can be easily converted to anatase TiO_2_ by a subsequent calcination at 400 ^o^C in air. The resultant anatase nanobelts show excellent performances when utilized as anode for LIBs. The current strategy can be extended to prepare other ultrathin nanomaterials and gives hints to nanostructure design for electrode materials.

## Results

The formation of the hydrogen titanate ultrathin nanobelts is based on a dissolution/precipitation mechanism between H_2_O_2_ and the Ti-based precursor. Hydrogen peroxide is inexpensive and environmentally benign, as the only degradation product of which is water. It is noted that many titanium precursors have been dissolved in aqueous H_2_O_2_ solution to produce certain peroxo complexes^40^,^41^; however, synthesis of nanobelts in the presence of H_2_O_2_ has not been reported yet^42^, let alone that of ultrathin nanobelts. Moreover, most titanium precursors, such as titanium alkoxides and titanium halides, are highly susceptible to water or even moisture. Once contacted with water, rapid hydrolysis occurs on the surface and TiO_2_ is precipitated in the form of spherical nanoparticles rather than that of 1D or 2D nanostructures. Thus, a key technical issue herein is to explore a Ti-based precursor which does not hydrolyze in water.

**Figure 2:**
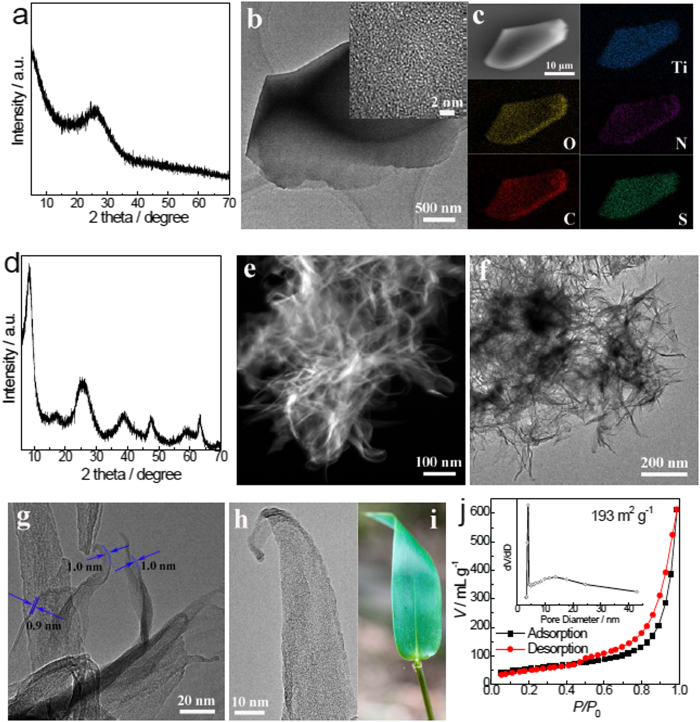
Characterization of precursor and titanate ultrathin nanobelts. (**a**) XRD pattern, (**b**) TEM image (inset: HRTEM image), and (**c**) EDS mapping of the precursor. (**d**) XRD pattern, (**e**) STEM image, (**f, g**) TEM images, and (**h**) HRTEM image of the as-prepared titanate ultrathin nanobelts. (**i**) Optical photograph of a bamboo leaf. (**j**) Nitrogen adsorption-desorption isotherm of the as-prepared titanate ultrathin nanobelts (inset: pore-size distribution calculated by BJH method from the desorption branch).

In current investigation, the black precursor achieved by the rapid pyrolysis is amorphous (Fig. 2a and Inset in Fig. 2b) and has a compact bulky morphology (Fig. 2b and Supplementary Fig. 1a), which remains almost unchanged after being immersed in water, either at ambient condition (Supplementary Fig. 1b) or at 160 ^o^C in a hydrothermal condition (Supplementary Fig. 1c), demonstrating its excellent stability in water. This water-stable feature is very important for a convenient and safe usage and storage in practice. The precursor contains uniformly distributed Ti, O, N, C, and S elements (Fig. 2c). The mass fraction of C, N, and H measured by an element analysis (Flash EA 1112, ThermoFinnigan) is 25.2%, 15.4%, and 2.8%, respectively. The precursor mainly contains Ti-O, O-H, C–C, C–N, N-H, O = C–O, Ti-S-O, and SO_4_^2−^ species (Supplementary Fig. 2 and Supplementary Fig. 3). After the reaction with H_2_O_2_ at ambient conditions, the precursor was converted to hydrogen titanate ultrathin nanobelts. Hydrogen titanates tend to form 2D morphologies because of their inherent lamellar structure. The room temperature synthesis further guarantees an ultrathin thickness of the titanate. Contrast to low yield of dominant liquid exfoliation route for preparation of ultrathin layered materials^25^, the precursor completely converts to hydrogen titanate ultrathin nanobelts in the present synthesis strategy. When compared with hydrothermal/solvothermal reactions widely adopted for the synthesis of most titanate nanostructures, the present route is energy-efficient as it demands no heating or external mechanical activations. The pyrolysis procedure for the precursor preparation is simple and rapid. The main external thermal energy input is for dehydration, which is the most time-consuming procedure. The pyrolysis process that finally achieved the black precursor is completed within just 2 min.

The phase and lamellar structures of the titanate were characterized by X-ray diffraction (XRD). All the diffraction peaks of the as-prepared sample (Fig. 2d) can be indexed to orthorhombic hydrogen titanate (H_2_Ti_2_O_5_.H_2_O, JCPDS card 47–0124). The broadening of the reflection peaks indicates small grains in the as-prepared titanate. The strong Bragg peak at 2*θ* = 8.5^o^ corresponds to the lamellar structure with an interlayer distance of ca. 1 nm. This interlayer distance is larger than that of hydrogen titanate (H_2_Ti_2_O_5_.H_2_O), which may be attributed to the intercalation of nitrogen species and ammonium ions (Supplementary Fig. 4)^37^.

The as-prepared titanate exhibits a 1D-like architecture with abundant pores, as shown in the scanning transmission electron microscopy (STEM, Fig. 2e) and transmission electron microscopy (TEM, Fig. 2f) images. A closer observation reveals the nanobelt with a sharp tip (Fig. 2g,h), which looks like a bamboo leaf as a whole (Fig. 2i). The tips are curved and the edges are rolled up (Fig. 2e–h) because of the surface tension, which is commonly observed in graphene^43^,^44^. This suggests the ultrathin and flexible characteristics for the present titanate nanobelts. Many single-layered hydrogen titanates with a thickness of *ca*. 1 nm can be observed in Fig. 2g,h, which is in accordance with the XRD result. Some stacking nanobelts are also observed (Supplementary Fig. 5). The thickness of most nanobelts is estimated to be 1– 2nm (Fig. 2g,h, and Supplementary Fig. 5). The nanobelts sway, gather, shrink, and thicken under electron beam irradiation during TEM observations, which can be attributed to its ultrathin and flexible features and the instability of hydrogen titanate under the electron beam. It is previously observed that hydrogen titanate decomposed easily to anatase under focused electron beam irradiation^45^. This can explain the inconsistency that the titanate is crystallized as revealed by XRD (Fig. 2d) but shows no crystals in TEM observations (Fig. 2g,h). The titanate nanobelts possess a high specific surface area of 193 cm^3^g^−1^ and large total pore volume of 0.949 cm^3^g^−1^ (Fig. 2j). It is worth mentioning that the ultrathin nanobelts can be obtained in a wide range of H_2_O_2_ amount (20–800 mL for one gram of precursor, Supplementary Fig. 6), which indicates a high reliability of the present synthesis strategy. The increased and then decreased Ti(IV) concentration in the solution (Supplementary Fig. 7) confirms a dissolution/precipitation mechanism for the formation of titanate nanobelts when immersing the black titanium-based precursor in aqueous H_2_O_2_ solution.

**Figure 3:**
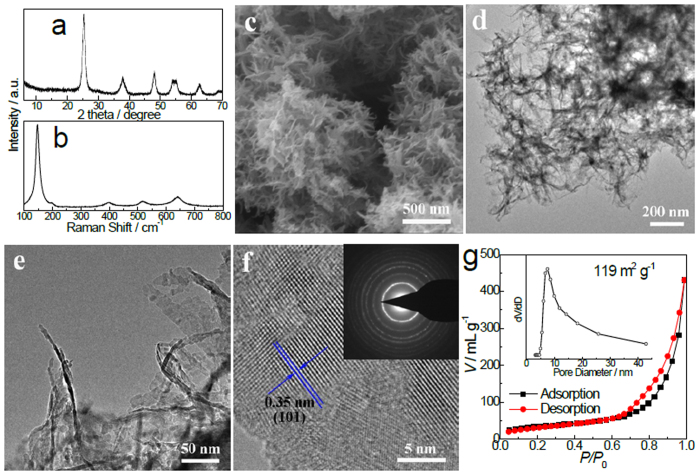
Characterization of anatase TiO_2_ ultrathin nanobelts. (**a**) XRD pattern. (**b**) Raman spectrum. (**c**) SEM image. (**d,e**) TEM images. (**f**) HRTEM image (inset: SAED pattern). (**g**) Nitrogen adsorption-desorption isotherm (inset: pore-size distribution calculated by BJH method from the desorption branch).

Phase-pure anatase TiO_2_, as verified by the XRD pattern (Fig. 3a) and Raman spectrum (Fig. 3b), can be obtained by a subsequent calcination of the as-prepared titanate in air, with the ultrathin nanobelts architecture well reserved (Fig. 3c–e). The transition temperature (400 ^o^C) from titanate to anatase here is lower than that of titanate obtained by hydrothermal reactions followed by a subsequent proton-exchange procedure^26^,^27^,^28^,^29^,^30^. Gentili et al. argued that a lower Na/Ti ratio in titanates was in favor of its transition to anatase^46^. Thus, the relatively low transition temperature could be attributed to the sodium-free feature for the titanate achieved in the current investigation. The high-resolution TEM (HRTEM) image of a nanobelt (Fig. 3f) exhibits a recognizable lattice spacing of 0.35 nm, corresponding to the (101) atomic plane of anatase TiO_2_. The selected area electron diffraction (SAED) pattern (Inset in Fig. 3f) further confirms that the nanobelt is in anatase polycrystalline. The thickness of anatase nanobelts is typically below 5 nm (Fig. 3e), slightly larger than that of the as-synthesized titanate because of the phase transition and grain growth during the calcination in air. The specific surface area and total pore volume of the anatase TiO_2_ nanobelts is determined to be 119 m^2^g^−1^ and 0.666 cm^3^g^−1^ (Fig. 3g), respectively.

**Figure 4:**
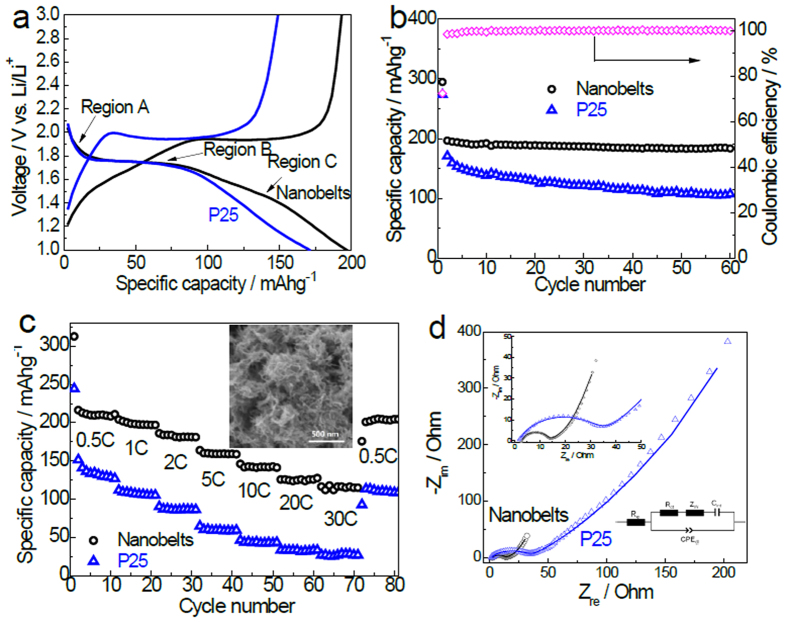
Electrochemical measurements of the anatase TiO_2_ ultrathin nanobelts and commercial P25 TiO_2_ nanoparticles. (**a**) The second discharge-charge profiles at the current rate of 1 C. (**b**) Cycling performance at 1 C. (**c**) Rate performance (inset: SEM image of the electrode after rate performance test). (**d**) Electrochemical impedance spectra measured in the open circuit potential over the frequency range from 100 kHz to 0.01 Hz (insets: enlarged view of the semicircle regions and the equivalent circuit), the specimens are those after the rate performance testing as illustrated in (**c**). The fitting results are represented by the solid lines.

The electrochemical performance of the anatase TiO_2_ ultrathin nanobelts was evaluated in coin type lithium half-cells using Li foil as counter electrode and reference electrode at the room temperature. Based on the shape, the discharge curve of the TiO_2_ nanobelts (Fig. 4a) can be divided into three regions^46^: i) a voltage drop from the open circuit voltage to ca. 1.75 V, which is related to the formation of the solid–solution Li_*x*_TiO_2_; ii) a voltage plateau at ca. 1.75 V, which indicates a phase equilibrium between the anorthorhombic Li_0.5_TiO_2_ phase and the Li-poor tetragonal Li_*x*_TiO_2_ phase; iii) a sloped region from 1.75 V to 1.0 V, which is attributed to the Li-ion insertion process into the surface layer of nano-sized TiO_2_ under the external force of the electric field. The high operating potential enables the batteries to be charged at high rates with high safety. The reaction in a TiO_2_/Li half-cell is proposed to be,


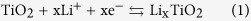


Here, the maximum insertion coefficient *x* is determined to be only ~0.5 (corresponding to a capacity of 167.5 mAhg^−1^) in bulk TiO_2_ due to the strong repulsive force between Li ions when the insertion ratio is greater than 0.5 [Refs 24,47]. Both theoretical simulations and experimental results demonstrated that the theoretical capacity of titania-based electrodes can be dramatically improved when certain nanostructures were employed^5^,^6^,^11^. The nanobelts exhibit an enhanced specific capacity at 1 C (170 mAg^−1^) from 171 to 197 mAhg^−1^ when compared with Degussa P25, (a benchmark TiO_2_ nanoparticles) in the second cycle, as shown in Fig. 4a,b. Moreover, the nanobelts demonstrate an excellent cycling stability (Fig. 4b), which is much better than that of P25. The irreversible capacity in the 1st cycle is assigned to the non-reversible Li intercalation or the reaction between the electrolyte and the anatase surface^13^,^14^,^15^,^16^,^17^,^18^,^19^, which is common for most anode materials, especially for nanostructured materials. Fortunately, the irreversible capacity loss in the first cycle can be effectively and conveniently mitigated by a butyllithium (C_2_H_5_OLi) treatment, or nano-sized Li powder incorporation^48^,^49^. After 5 cycles, the columbic efficiency kept a stable value of over 99.5%, suggesting a highly reversible electrochemical reaction.

As shown in Fig. 4c, the anatase TiO_2_ ultrathin nanobelts deliver a discharge capacity of ca. 216, 204, 186, 164, 146, 126, and 116 mAhg^−1^ at a current rate of 0.5 C, 1 C, 2 C, 5 C, 10 C, 20 C, and 30 C, respectively. When the current is returned to 0.5 C, the anatase TiO_2_ ultrathin nanobelts resumes its capacity of 204 mAhg^−1^ after 80 cycles at different current densities, suggesting its excellent rate performance and good cycling stability. On the contrary, the P25 only displays 151, 112, 90, 65, 46, 33, and 27 mAhg^−1^ at 0.5 C, 1 C, 2 C, 5 C, 10 C, 20 C, and 30 C, respectively, which is much lower than that of the anatase TiO_2_ ultrathin nanobelts. It took only 2 min at 30 C to charge the nanobelts to 116 mAhg^−1^ while charging the P25 to 112 mAhg^−1^ took a much longer time of 1 h at 1 C (Fig. 4c). At 30 C, the nanobelts illustrate a 330% capacity improvement over the P25 (Fig. 4c). The electrochemical performance of anatase TiO_2_ ultrathin nanobelts is not only better than TiO_2_ nanobelts obtained by a common alkaline hydrothermal route^50^,^51^ but also better than most of high performance TiO_2_ anodes^17^,^19^,^24^,^52^,^53^,^54^,^55^,^56^,^57^,^58^. The high electrochemical activity and low-cost preparation make the TiO_2_ ultrathin nanobelts a promising anode material for high performance and safe LIBs.

## Discussion

The excellent electrochemical performance is mainly ascribed to its unique nanostructure: a) the ultrathin thickness greatly shortens the diffusion distance of Li ions and the 1D-like structure possesses facile charge transport along the longitudinal dimension; b) the porous structure allows efficient ingress and infiltration of the electrolyte into the electrode, enabling a rapid ion transport, and provides abundant electrolyte/electrode contact area for charge transfer; c) the porous and flexible nanostructure effectively accommodates the volume/strain changes during the charge-discharge process. After the rate performance test for 80 cycles, the original ultrathin nanobelt structures can be well-retained (Inset in Fig. 4c), demonstrating the structure stability during charge-discharge cycles. The special composition of the nanobelts may also contribute to the excellent electrochemical performance. The X-ray photoelectron spectrum (XPS) N 1s signal ranging from 398 to 402 eV (Supplementary Fig. 8a) on the surface of the TiO_2_ nanobelts can be attributed to major interstitial nitrogen (N_inter_) and minor chemically adsorbed nitrogen (N_ad_) species^59^. The S 2p peak at 168.4 eV (Supplementary Fig. 8b) is assigned to sulfur species in SO_4_^2−^ formed on the TiO_2_ nanobelts surface^59^. The N and S are *in-situ* doped to TiO_2_ nanobelts from the N-/S-containing precursor, resulting in a pale yellow color and an enhanced visible light absorption of the TiO_2_ nanobelts (Supplementary Fig. 9). Recently, Jiao et al. reported that N and S co-doping could improve lithium storage capability of TiO_2_^59^. The exact effect of surface species and crystal defects on lithium storage performance of the present TiO_2_ nanobelts requires further investigations. Nevertheless, the nanobelts indeed show obviously enhanced charge transfer when compared with that of P25. In the electrochemical impedance spectroscopy (EIS) spectra illustrated in Fig. 4d, the intercept on Z_real_ axis in the high frequency region represents the resistance of electrolyte^60^. The depressed semicircle in the high-medium frequency region is assigned to the charge transfer process. The straight line at a lower frequency region represents the typical Warburg behavior, which is associated with the diffusion of Li ions in the electrodes. The impedance spectra are fitted to the proposed equivalent circuit (Inset in Fig. 4d), in which the equivalent circuit, R_e_, R_ct_, Z_w_, CPE_dl_, and C_int_ represent the electrolyte resistance, the charge-transfer resistance, the Warburg impedance, the double-layer capacitance, and the intercalation capacitance, respectively^61^,^62^. The R_e_ of nanobelts and P25 is determined to be ca. 2.8 Ω and 1.9 Ω, respectively. The R_ct_ of nanobelts and P25 is determined to be ca. 10.5 Ω and 33.0 Ω, respectively. Although the electrolyte resistance of nanobelts is slightly larger than that of P25, the nanobelts show much smaller charge transfer resistance (R_ct_) than that of P25. The nanobelts have larger slopes and shorter lines in the low frequency region, implying a faster Li^+^ diffusion rates and smaller variation of diffusion paths^63^. Thus, the small charge-transfer resistance on the electrode/electrolyte interface and fast Li^+^ diffusion contribute to the excellent electrochemical performance of the ultrathin nanobelts^62^.

**Figure 5:**
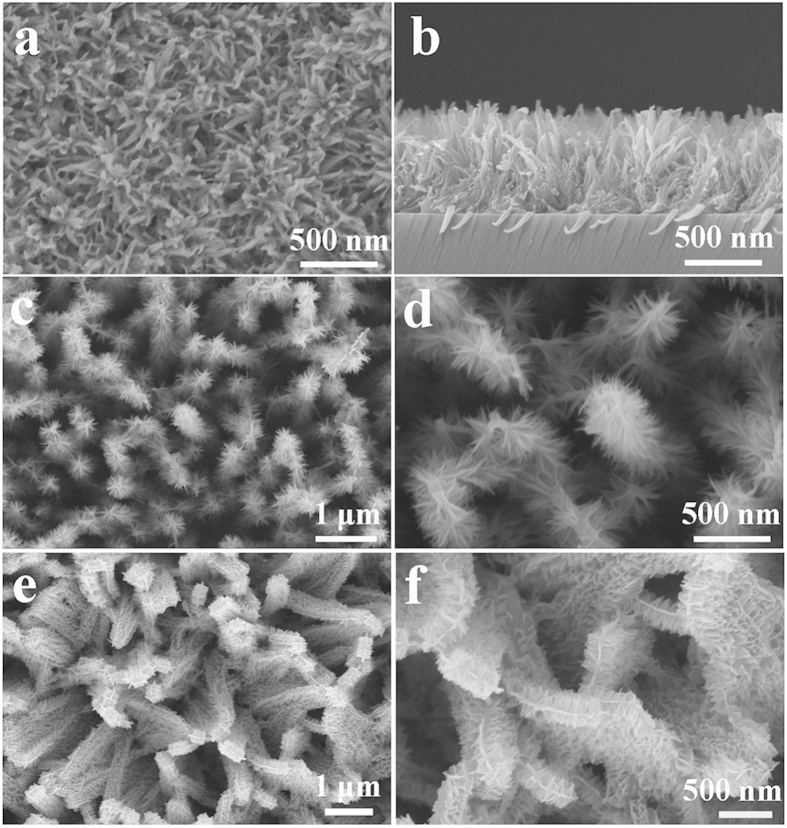
Characterization of titanate array films. (**a**) Top view and (**b**) cross sectional SEM images of titanate arrays precipitated on glass substrates. (**c,d**) SEM images of the core-shell branched nanowire arrays. (**e,f**) SEM images of the core-shell branched nanobelt arrays.

Interestingly, the synthesis strategy described here is capable of constructing titanate nanobelt arrays (Fig. 5a,b) and branched core-shell arrays (Fig. 5c–f, namely ultrathin nanobelts deposited on anatase TiO_2_ nanowire or nanobelt arrays) on various plane and complex substrates without the assistance of any seed layers. TiO_2_ arrays with various 1D/3D nanostructures have potential applications in regions of solar energy conversion, energy storage, and wettability control^64^,^65^,^66^. It is noted that the growth of nanostructured hydrogen titanate arrays via alkaline hydrothermal route is always limited to metallic Ti substrates^32^, or other substrates with a Ti coating^35^. Moreover, composition decoration can be conveniently achieved by this method (Supplementary Figs. 9–14), which may exhibit enhanced properties in lithium storage^67^ and photoelectrochemical water splitting^68^,^69^.

In summary, hydrogen titanate ultrathin nanobelts were synthesized on a large scale at room temperature by a facile strategy based on a H_2_O_2_-asisted dissolution/precipitation process, with an appropriately designed water-proof Ti-based precursor. Anatase TiO_2_ can be obtained by a calcination of the as-synthesized titanate at a relatively low temperature of 400 ^o^C in air, with the ultrathin nanobelts architecture well-reserved. The unique nanostructures and appropriate composition endowed the anatase TiO_2_ nanobelts excellent performances in lithium storage. The high electrochemical activity and low-cost preparation make the TiO_2_ ultrathin nanobelts a promising anode material for fast and safe LIBs. This synthesis route is also convenient for metal decoration, as well as constructions of 1D/3D TiO_2_ arrays on arbitrary substrates. The current strategy can be extended to prepare other ultrathin metal oxides, which may find wide applications in regions of catalysis, energy conversion, and energy storage.

## Methods

### Synthesis

All regents were of analytical grade and used as received without further purifications. 1.75 g glycine (C_2_H_5_NO_2_), 1.25 g titanium oxysulfate-sulfuric acid hydrate (Aladdin), and 0.6 mL nitric acid (65 wt. %) were added in 10 mL deionized water in a 100 mL crucible, which was transferred to a preheated furnace maintained at 400 °C for ca. 15 min to obtain a black precursor after ultrasounded for 10 min and stirred for 1 h. 0.5 g black precursor was then added into 400 mL H_2_O_2_ (30 wt. %) and stored at room temperature for 72 h to obtain hydrogen titanates, which were converted to anatase TiO_2_ counterparts after calcination in air at 400 ^o^C for 1 h with a heating rate of 1 ^o^C min^−1^.

### Characterizations

X-ray diffraction (XRD) measurements were conducted on a XRD-6000 diffractometer (SHIMADZU) with a Cu Kα radiation, operated at 40 kV, 40 mA (λ = 0.15406 nm). The powder morphology was observed using a field emission scanning electron microscopy (FE-SEM, Hitachi S-4800, Tokyo, Japan, with EDS capabilities), together with a transmission electron microscopy (TEM, FEI-F20, FEI, USA) working at 200 kV. The X-ray photoelectron spectra (XPS) characterization was carried out on an Escalab 250Xi system (Thermo Fisher Scientific). The binding energy (BE) was calibrated by using the containment carbon (C 1 s = 284.6 eV). The Raman spectra were taken on a LabRamHRUV (JDbin-yvon) Raman spectrometer, using the 514 nm line as the excitation source. The Brunauer-Emmett-Teller (BET) approach using adsorption data was utilized to determine the specific surface area. The sample was degassed at 150 °C for 14 h to remove physisorbed gases prior to the measurement. Titanium concentrations of the reaction solution sampled at various time intervals were measured by ICP-MS (XSENIES).

### Lithium storage test

The working electrodes were prepared by a slurry coating procedure. The slurry consisted of 70 wt. % TiO_2_ powders, 20 wt. % acetylene black, and 10 wt. % polyvinylidene fluoride (PVDF) dispersed in N-methyl pyrrolidinone (NMP), and was coated on a copper foil, which acted as a current collector. The film was dried at 90 °C for 20 h in vacuum. The cells were assembled in an argon-filled glove box using Li foil as a counter electrode and polypropylene (PP) film (Celgard 2300) as a separator. The electrolyte was 1 M LiPF_6_ in a 50:50 (w/w) mixture of ethylene carbonate (EC) and diethyl carbonate (DEC). The charge-discharge tests were conducted on a LAND 2001A system. The electrochemical impedance spectroscopy (EIS) measurements were performed on a CHI660D electrochemical workstation.

## Additional Information

**How to cite this article**: Wen, W. *et al.* Anatase TiO_2_ ultrathin nanobelts derived from room-temperature-synthesized titanates for fast and safe lithium storage. *Sci. Rep.* **5**, 11804; doi: 10.1038/srep11804 (2015).

## Supplementary Material

Supplementary Information

## Figures and Tables

**Figure 1:**
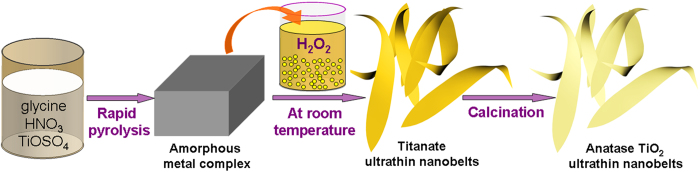
A schematic illustration showing the novel synthesis procedure of anatase TiO_2_ ultrathin nanobelts. Firstly, an amorphous precursor was prepared via a rapid pyrolysis process. Then, room temperature treatment of the amorphous precursor with H_2_O_2_ resulted in the formation of hydrogen titanate ultrathin nanobelts. Finally, anatase TiO_2_ ultrathin nanobelts were obtained by a calcination of the hydrogen titanate ultrathin nanobelts.
