# Supplementary material for: Anatase TiO2 ultrathin nanobelts derived from room-temperature-synthesized titanates for fast and safe lithium storage
Source: Sci Rep. 2015 Jul 2;5:11804. doi: 10.1038/srep11804 (PMC4488874; doi:10.1038/srep11804)
Supplement: Supplementary Information [file srep11804-s1.pdf]

Supporting Information

For

Anatase TiO_2 ultrathin nanobelts derived from room-temperature-synthesized titanates for fast and safe lithium storage

Wei Wen[a,c], Jin-Ming Wu[a*], Yin-Zhu Jiang[a], Sheng-Lan Yu[a], Jun-qiang Bai[a], Min-Hua Cao[b], Jie Cui[d]

[a] State Key Laboratory of Silicon Materials, Key Laboratory of Advanced Materials and Applications for Batteries of Zhejiang Province, and school of Materials Science and Engineering, Zhejiang University, Hangzhou 310027, P. R. China.

[b] Key Laboratory of Cluster Science, Ministry of Education of China, and Department of Chemistry, Beijing Institute of Technology, Beijing 100081, P. R. China.

[c] College of Mechanical and Electrical Engineering, Hainan University, Haikou 570228, P. R. China.

[d] Department of Physics, Wenzhou University, Wenzhou 325035, P. R. China.

Additional materials synthesis

1. Synthesis of metal doped anatase TiO_2 ultrathin nanobelts.

1.75 g glycine ($C_2H_5NO_2$), 1.25 g titanium oxysulfate-sulfuric acid hydrate (Aladdin), 0.6 mL nitric acid (65%), and 0.15 mmol metal nitrate ($M(NO_3)_2$, M = Co, Ni, Mn, Zn) were added in 10 mL deionized water in a 100 mL crucible, which, after ultrasounded for 10 min and stirred for 1 h, was transferred to a preheated furnace maintained at 400 ºC for ca. 15 min to obtain a black precursor. 0.5 g black precursor was then added into 400 mL H_2O_2 (30 wt%) and stored at room temperature for 72 h to obtain hydrogen titanates. The hydrogen titanates doped with various metal ions were converted to anatase TiO_2 counterpart after calcination in air at 400 ^oC for 1 h with a heating rate of 1 $^oC\ min^{-1}$.

2. Synthesis of titanate nanobelt arrays on glass substrates.

The solution after reactions of 0.5 g black precursor and 50 mL H_2O_2 (30 wt%) at room temperature for 72 h was subjected to centrifugation to recover the titanate precipitations. The residual solution, labeled as precursor solution, was then used for thin film depositions. Cleaned glass substrates with $2.5 \times 2.5\ cm^2$ in size was then immersed in 15 mL of the precursor solution at 80 ^oC for 24 h. The morphology of the films is shown in Figure 5a, b in the main text.

3. Synthesis of branched nanowire@nanobelt core-shell structures on Ti substrate.

3.1 Synthesis of TiO_2 nanowire arrays.

Synthesis of the anatase TiO_2 nanowire arrays with diameter of 80 nm on Ti substrates was similar to a previously reported procedure[1]. A piece of titanium foil (2.5 × 2.5 cm^2) was pickled for 2 min at 60 ^oC in a 1:3:6 (in volume) mixture of aqueous solutions of HF (55 wt %), HNO_3 (63 wt %), and distilled water, followed by cleaning with water in an ultrasonic bath. The cleaned Ti foil was then placed in a 50 ml Teflon-lined stainless steel autoclave filled with 35 mL of 1.25 M NaOH in aqueous solution. The sealed autoclave was put in an oven at 220 °C for 20 h. Then, the titanium foil covered with $Na_2Ti_2O_5 \cdot H_2O$ nanowires was immersed in 0.1 M HCl solution for 40 min and this process was repeated for 3 times to replace Na^+ with H^+, resulting in the formation of $H_2Ti_2O_5 \cdot H_2O$ nanowire arrays on Ti foil. The sample was removed from HCl solution and rinsed with water and ethanol, then dried at 80 °C. The anatase TiO_2 nanowire arrays were obtained after a calcination of the $H_2Ti_2O_5 \cdot H_2O$ nanowire arrays at 550 °C for 3 h in air with a heating rate of 2 ºC min^{-1}. Finally, The Ti foil covered with TiO_2 nanowire arrays was ultrasounded for 90 s to remove the weakly bonded nanowires on surface.

3.2 Growth of nanobelt branches on the TiO_2 nanowire arrays.

The Ti foil 2.5 × 2.5 cm^2 in size, covered with the anatase TiO_2 nanowire arrays, was immersed in 15 mL precursor solution at 80 ^oC for 3 h. The morphology of the resultant core-shell structures is shown in Figure 5c, d in the main text.

4. Synthesis of branched nanobelt@nanobelt core-shell structures on Ti substrate.

4.1 Synthesis of TiO_2 nanobelt arrays.

A cleaned Ti foil (2.5 × 2.5 cm^2) was placed in a 50 ml Teflon-lined stainless steel autoclave filled with 40 mL of 5 M NaOH in aqueous solution. The sealed autoclave was put in an oven at 180 °C for 24 h. Then, the titanium foil covered with $Na_2Ti_2O_5 \cdot H_2O$ nanobelts was immersed in 0.1 M HCl solution for 40 min and this process was repeated for 3 times to replace Na^+ with H^+, resulting in the formation of $H_2Ti_2O_5 \cdot H_2O$ nanobelt arrays on Ti foil. The sample was removed from HCl solution and rinsed with water and ethanol, then dried at 80 °C. The anatase TiO_2 nanobelt arrays were obtained after a calcination of the $H_2Ti_2O_5 \cdot H_2O$ nanobelt arrays at 450 °C for 1 h in air with a heating rate of 2 ºC min^{-1}.

4.2 Growth of nanobelt branches on the TiO_2 nanobelt arrays.

The Ti foil 0.7 × 0.7 cm^2 in size, covered with the anatase TiO_2 nanobelt arrays, was immersed in 5 mL precursor solution at 80 ^oC for 3 h. The morphology of the resultant product is shown in Figure 5e, f in the main text.

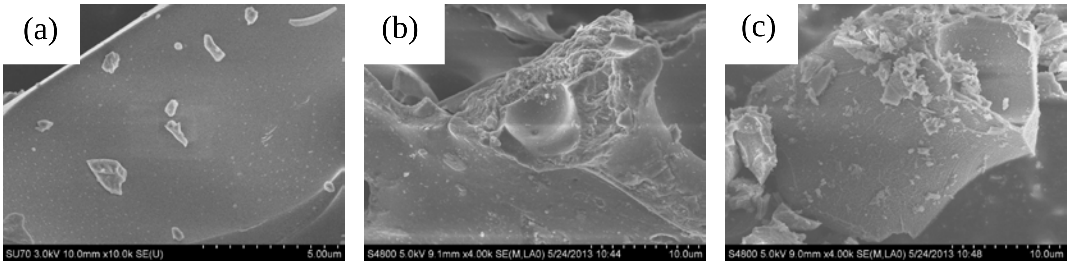

Figure 1. **SEM images of the initial precursor and those suffered by different treatments.** (a) SEM image of the precursor. (b) SEM image of the precursor after immersion in deionized water (0.5 g precursor in 400 mL water) at room temperature for 72 h. (c) SEM image of the precursor after immersion in deionized water at 160 ^{o}C for 12 h in an autoclave (0.1 g precursor in 40 mL water, 80% filled).

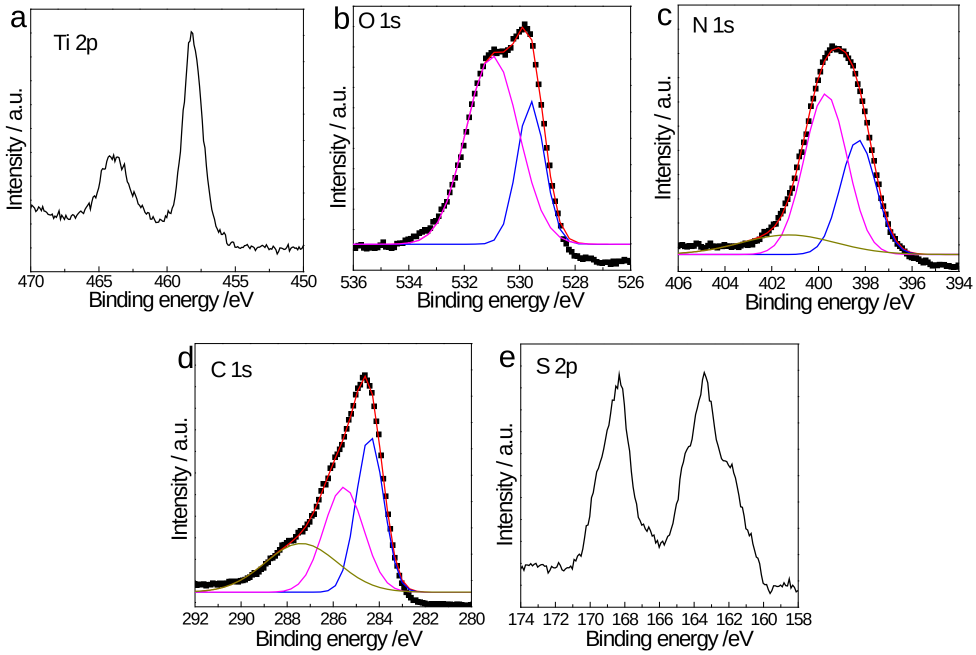

Figure 2 High-resolution XPS spectra of the precursor: (**a**) Ti 2p, (**b**) O 1s, (**c**) N 1s, (**d**) C 1s, and (**e**) S 2p.

X-ray photoelectron spectra (XPS) were carried out to investigate the composition of the titanium-based complex and the results are shown in Fig. 2. The Ti 2p spectrum (Fig. 2a) shows two peaks of the $2p_{3/2}$ and $2p_{1/2}$ doublet. The Ti $2p_{3/2}$ and $2p_{1/2}$ binding energies at ca. 458.2 and 464.0 eV are in good agreement with the energies of the photoelectrons of Ti^{4+} [Ref. 2]. The O 1s (Fig. 2b) at 529.6 eV is attributed to O^{2-} [Ref. 2], and the peak at 531.1 eV corresponds to covalent bonds of O with H or/and C [Ref. 3]. The N 1s spectrum (Fig. 2c) can be fitted to three peaks at 398.3, 399.7, and 401.2 eV, which may correspond to sp^3 C-N bond[4], N-H bond[5], and chemically absorbed nitrogen species[6,7], respectively. The C 1s spectrum (Fig. 2d) can be fitted into three peaks around 284.5, 285.6, and 287.4 eV, which is assigned to the C–C, C–OH/C–N, and O=C–O bond, respectively[8,9]. The S 2p peak (Fig. 2e) at 168.4 eV and 163.3 eV is assigned to sulfur species in SO_4^{2-} [Ref. 10] and Ti-S-O bond[11], respectively.

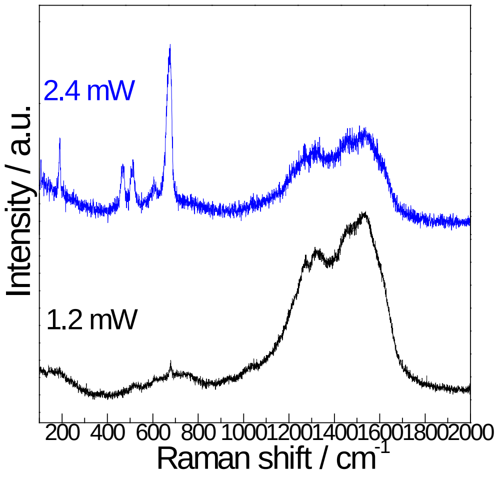

Figure 3 Raman spectra of the precursor obtained at different test conditions.

Figure 3 shows the Raman spectra of the precursor obtained at different laser powers. At a low laser power of 1.2 mW, there are two broad peaks at ca. 1315 and 1517 cm^{-1}, which may be attributed to attributed to the covalent bonds of C with C[12,13]. At a higher laser power of 2.4 mV, the intensity of the peaks at ca. 1315 and 1517 cm^{-1} decreases and it appears five sharp peaks at ca. 147, 196, 397, 516, 640 cm^{-1}, which is assigned to anatase TiO_2[14]. These results also suggest that the precursor can decompose and convert to anatase TiO_2 under laser irradiation with an appropriate power.

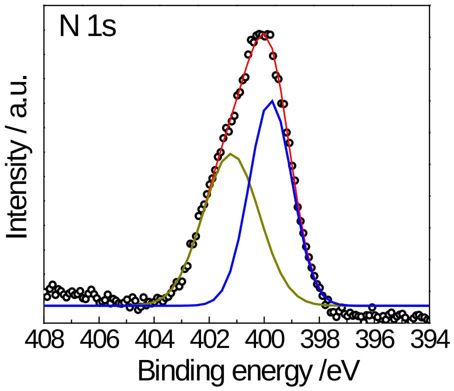

Figure 4. **N 1s high-resolution XPS spectrum of the hydrogen titanate ultrathin nanobelts.**

The binding energy at 399.7 eV and 401.2 eV is attributed to ammonium ions and absorbed nitrogen species, respectively[6].

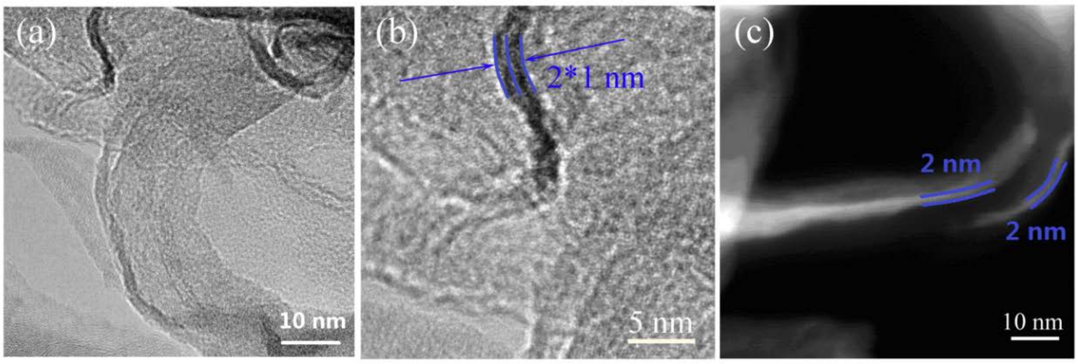

Figure 5. Characterization of the as-prepared titanate ultrathin nanobelts: (a, b) TEM images. **(c)** STEM image.

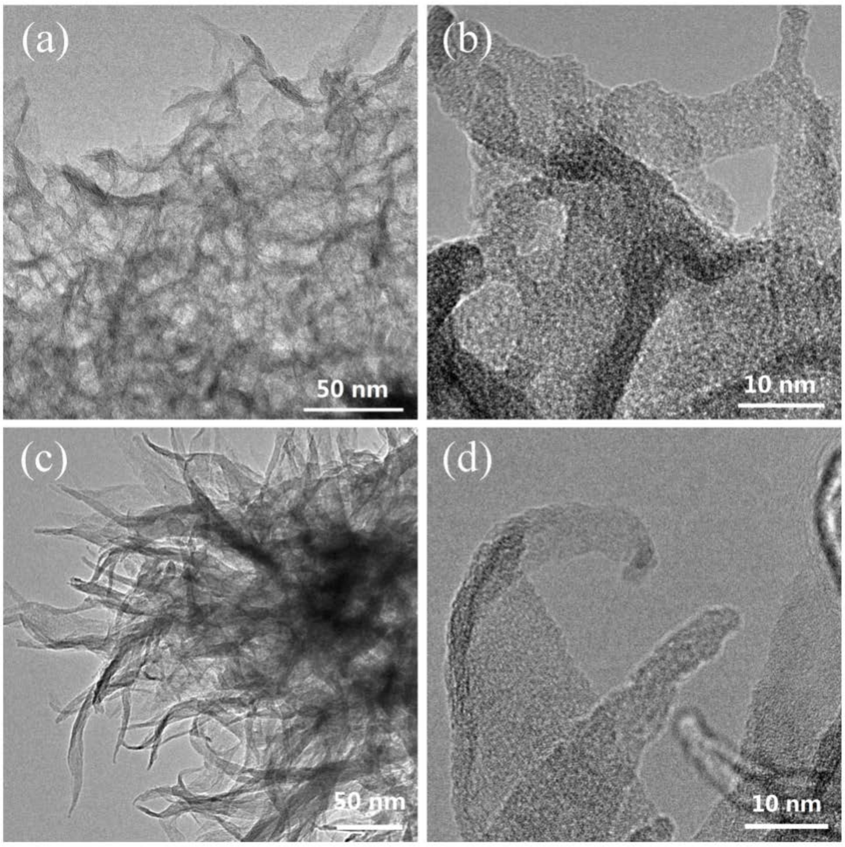

Figure 6. TEM images of products derived by the reaction of 0.5 g black precursor with different amount of H_2O_2 (30 wt%): (a, b) 10 mL and **(c, d)** 50 mL.

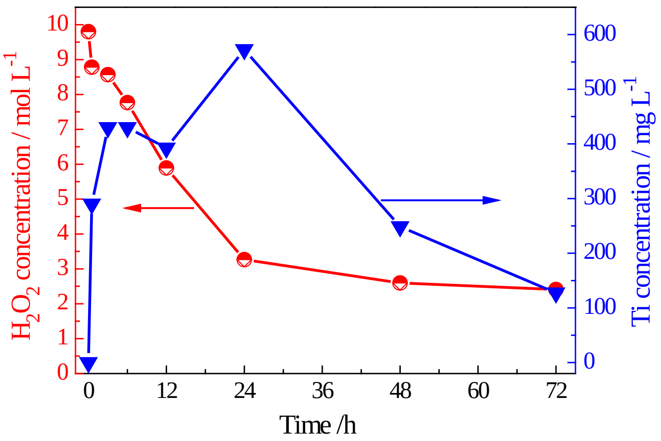

Figure 7. **Temporal evolutions of H_2O_2 concentration and Ti(IV) concentration.** The Ti(IV) and H_2O_2 concentration was measured by ICP-MS and titration method, respectively. 0.5 g black precursor was added into 50 mL H_2O_2 (30 wt%) and stored at room temperature in a time range of 0-72 h.

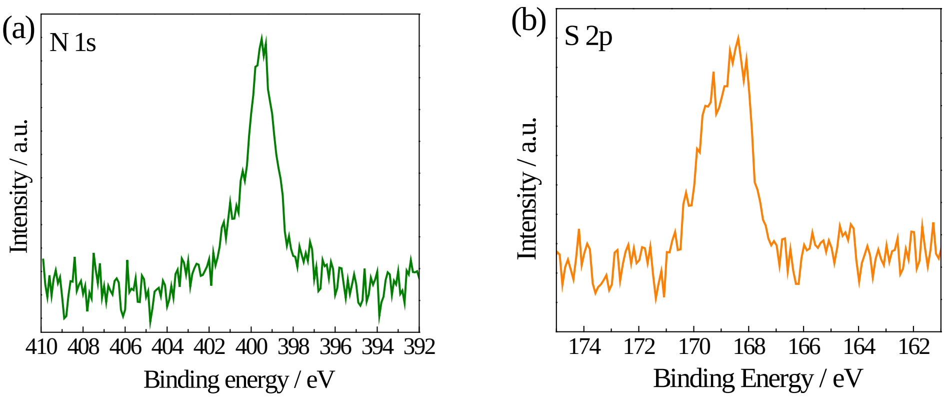

Figure 8. **High-resolution XPS spectra of the anatase TiO_2 ultrathin nanobelts**: (**a**) N 1s and (**b**) S 2p. The atomic ration of S/Ti and N/Ti on the surface of the anatase TiO_2 ultrathin nanobelts is determined to be 0.020 and 0.027 by XPS, respectively.

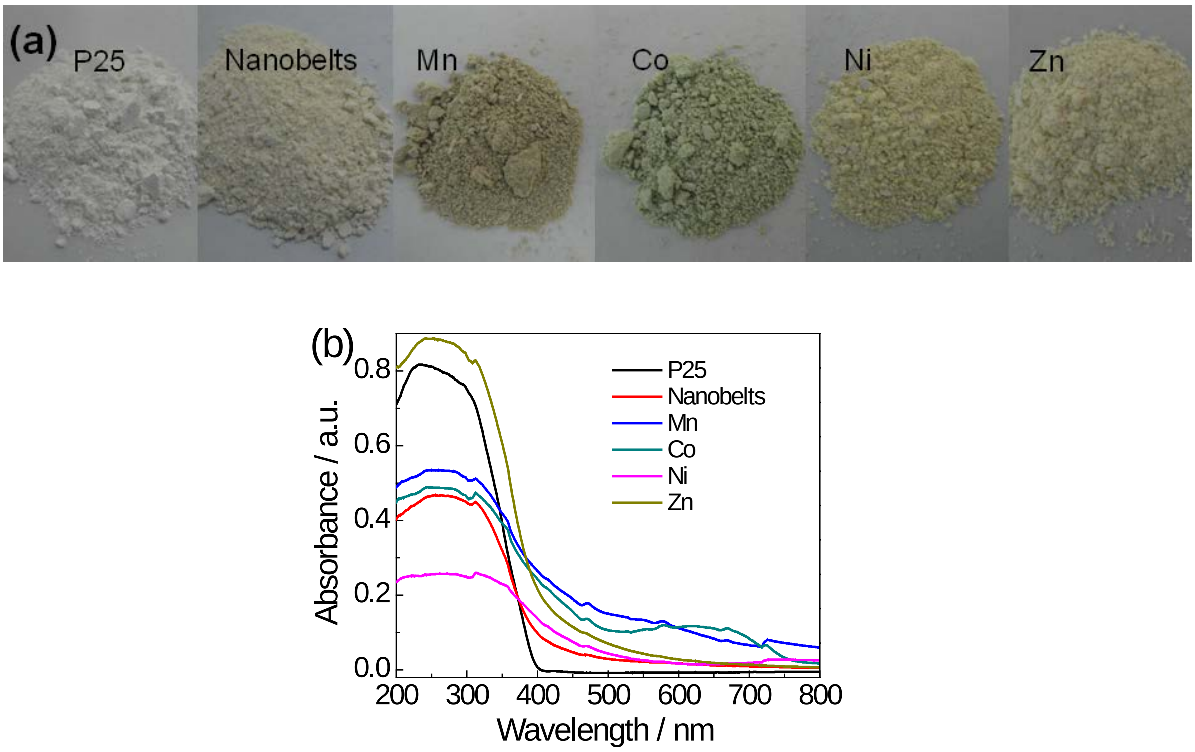

Figure 9. **Optical characterization of P25, anatase TiO_2 ultrathin nanobelts, and anatase TiO_2 ultrathin nanobelts decorated with different transition metal elements of Mn, Co, Ni, or Zn.** (**a**) Optical photographs. (**b**) UV- Visible diffuse reflectance spectra. All of the doped samples show enhanced visible light absorption. The spectra were collected using a Shimadzu UV-3600 PC ultraviolet visible near-infrared spectrophotometer with $BaSO_4$ as the reference material, and were converted from reflection to absorption according to the Kubelka-Munk method.

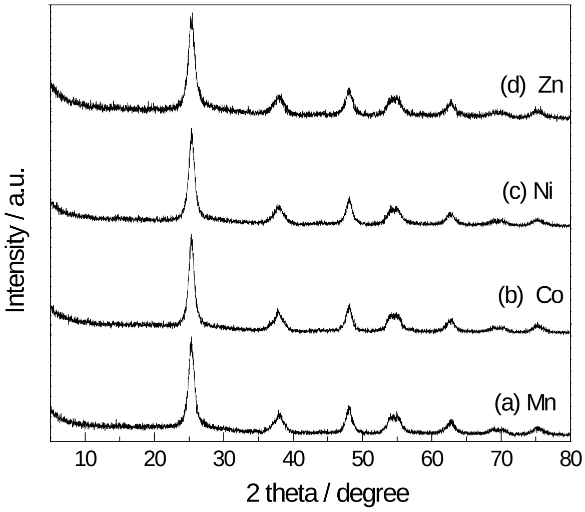

Figure 10. **XRD patterns of anatase TiO_2 ultrathin nanobelts decorated with different transition metal elements**: (**a**) Mn, (**b**) Co, (**c**) Ni, and (**d**) Zn. The anatase TiO_2 phase remains unaffected upon the various dopants.

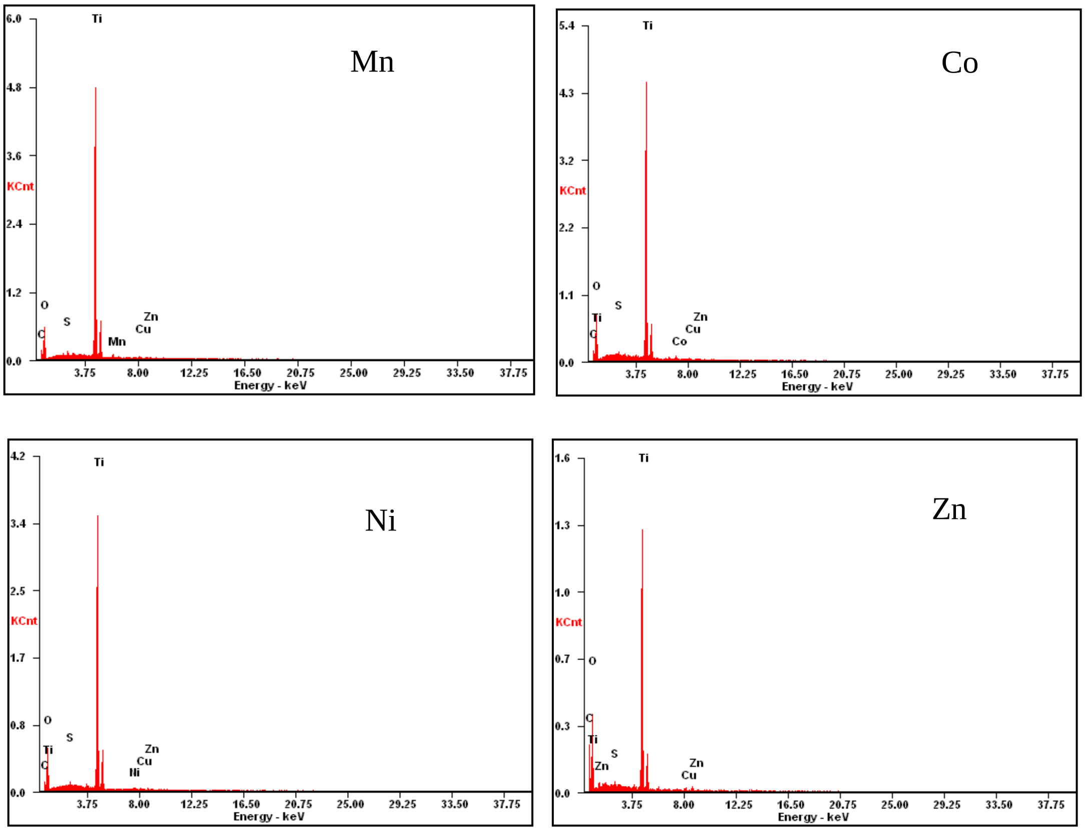

Figure 11. **EDS spectra of anatase TiO_2 ultrathin nanobelts decorated with different transition metal elements of Mn, Co, Ni, or Zn.**

The main C is from the tapes which adhere the powders to the SEM holder. The Cu and Zn is form the Cu-Zn alloy holder. The atomic ratio of Mn/Ti, Co/Ti and Ni/Ti is 0.018, 0.020 and 0.022, respectively. It is hard to estimate the atomic ratio of Zn/Ti in the Zn doped anatase TiO_2 ultrathin nanobelts because of disturbance from the SEM holder; however, the atomic ratio of Zn/Cu in the Zn doped sample is much higher than that of other three samples, which indicates the successful doping of Zn in the anatase TiO_2 ultrathin nanobelts.

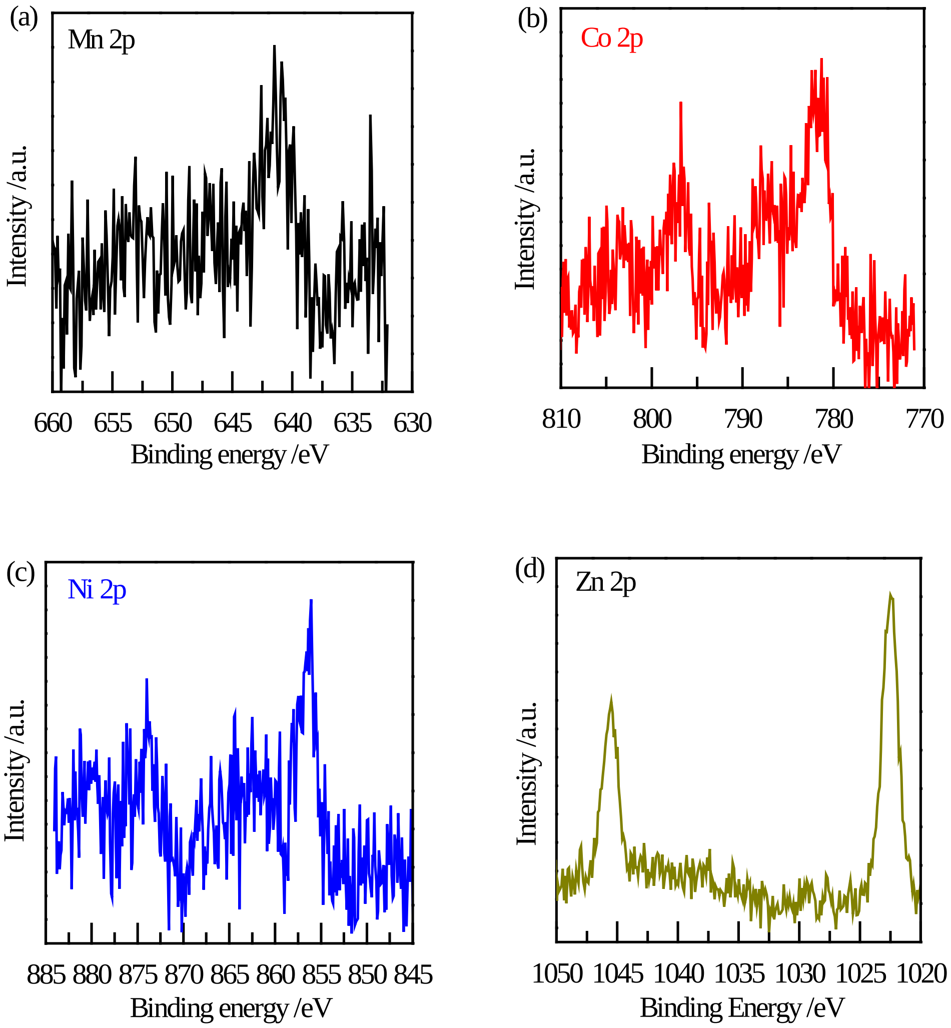

Figure 12. High-resolution XPS spectra of anatase TiO_2 ultrathin nanobelts decorated with different transition metal elements of Mn, Co, Ni, or Zn.

The high resolution XPS spectra indicate the presence of Mn, Co, Ni, and Zn in the corresponding decorated anatase TiO_2 nanobelts. The atomic ratio of Mn/Ti, Co/Ti, Ni/Ti, and Zn/Ti is 0.019, 0.041, 0.031, and 0.043 by XPS, respectively. The valence state of Mn, Co, Ni, and Zn is mainly +3, +2, +2, and +2, respectively[15-18].

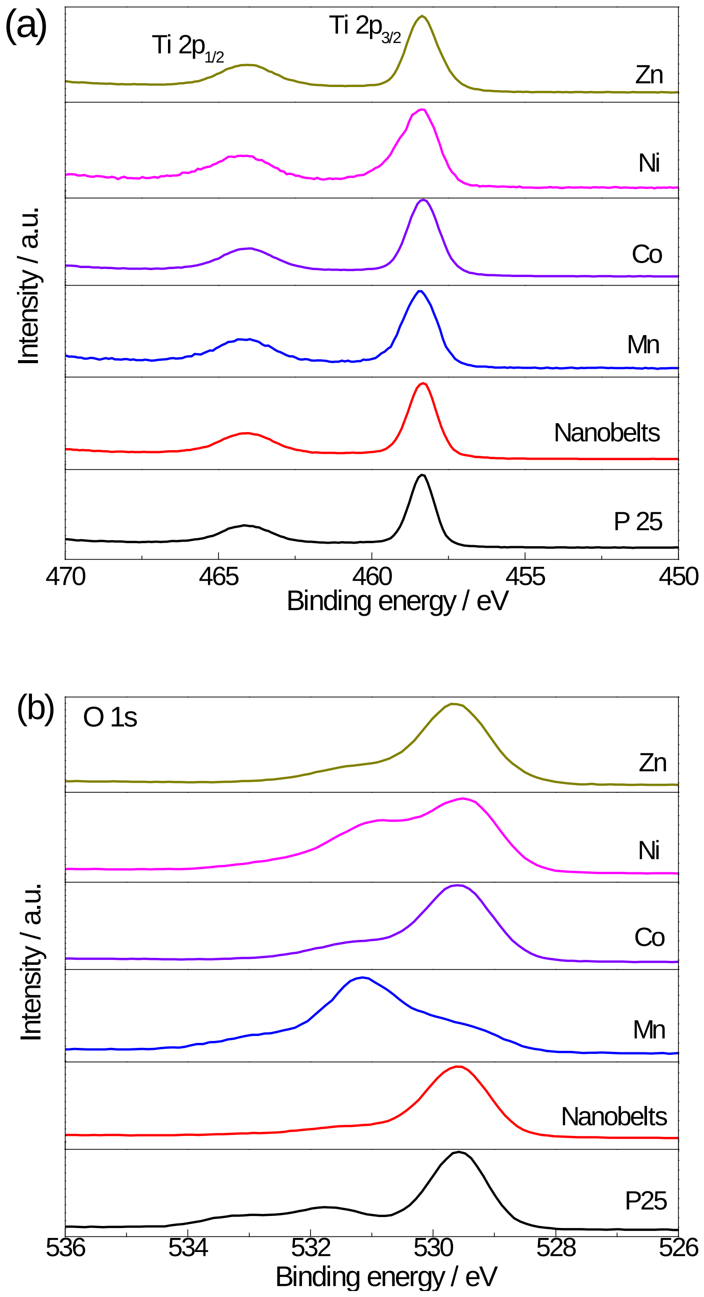

Figure 13. **High-resolution XPS spectra of P25, anatase TiO_2 ultrathin nanobelts, and anatase TiO_2 ultrathin nanobelts decorated with different transition metal elements (Mn, Co, Ni, or Zn): (a)** Ti 2p and **(b)** O 1s.

As shown in Fig. 13a, the Ti $2p_{3/2}$ and $2p_{1/2}$ binding energies of all above sample located at ca. 458.3 and 464.1 eV, respectively, which are in good agreement with the energies of the photoelectrons of Ti^{4+} [Ref. 2]. Comparing with the initial nanobelts and P25, the decorated samples show no obvious change in the binding energy of Ti 2p, which is consistent with the previous report[18]. The O 1s at 529.6 eV is attributed to lattice oxygen in TiO_2, and the peak at 531.2 eV corresponds to the oxygen in surface hydroxyl groups[19]. It can be seen that the samples decorated with Mn and Ni exhibited obviously increased hydroxyl groups (Fig. 13b).

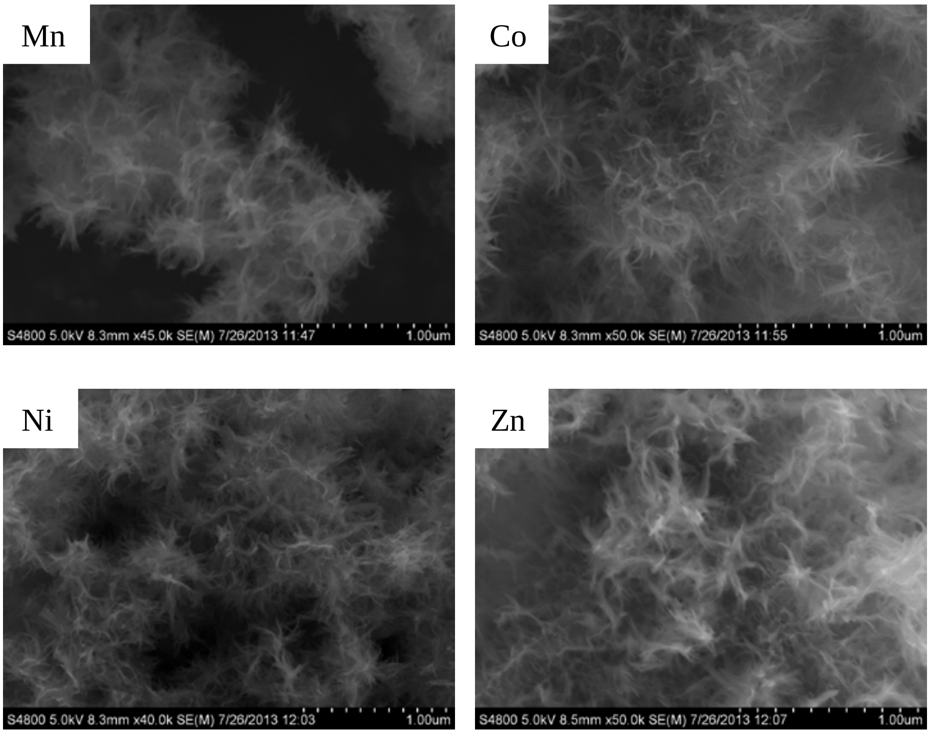

Figure 14. **SEM images of anatase TiO_2 ultrathin nanobelts decorated with different transition metal elements of Mn, Co, Ni, or Zn.** All of the decorated samples remain the morphology of ultrathin nanobelts unchanged.

References

1. Liao, J. Y., Higgins, D., Lui, G., Chabot, V., Xiao, X. & Chen, Z. Multifunctional TiO_2-C/MnO_2 core-double-shell nanowire arrays as high-performance 3D electrodes for lithium ion batteries. *Nano Lett.* **13**, 5467-5473 (2013).
2. Wu, J. M. & Qi, B. Low-temperature growth of rutile nanorod thin films and their photon-induced property. *J. Am. Ceram. Soc.* **91**, 3961-3970 (2008).
3. Shinde, S. S., Shinde, P. S., Oh, Y. W., Haranath, D., Bhosale, C. H. & Rajpure, K. Y. Investigation of structural, optical and luminescent properties of sprayed N-doped zinc oxide thin films. *J. Anal. Appl. Pyrolysis* **97**, 181-188 (2012).
4. Yamamoto, K., Koga, Y., Fujiwara, S., Kokai, F., Kleiman, J. I. & Kim, K. K. Carbon nitride thin films prepared by nitrogen ion assisted pulsed laser deposition of graphite using KrF excimer laser. *Thin Solid Films* **339**, 38-43 (1999).
5. Cao, P., Zhao, D. X., Zhang, J. Y., Shen, D. Z., Lu, Y. M., Yao, B., Li, B. H., Bai, Y. & Fan, X. W. Optical and electrical properties of p-type ZnO fabricated by NH_3 plasma post-treated ZnO thin Films. *Appl. Surf. Sci.* **254**, 2900-2904 (2008).
6. Liu, P., Zhang, H., Liu, H., Wang, Y., Yao, X., Zhu, G., Zhang, S. & Zhao, H. A facile vapor-phase hydrothermal method for direct growth of titanate nanotubes on a titanium substrate via a distinctive nanosheet roll-up mechanism. *J. Am. Chem. Soc.* **133**, 19032-19035 (2011).

7. Asahi, R., Morikawa, T., Ohwaki, T., Aoki, K. & Taga, Y. Visible-light photocatalysis in nitrogen-doped titanium oxides. *Science* **293**, 269-271 (2001).
8. Lai, L., Zhu, J., Li, Z., Yu, D. Y. W., Jiang, S., Cai, X., Yan, Q., Lam, Y. M., Shen, Z. & Lin, J. Co_3O_4/nitrogen modified graphene electrode as Li-ion battery anode with high reversible capacity and improved initial cycle performance. *Nano Energy* **3**, 134-143 (2014).
9. Sheng, Z. H., Shao, L., Chen, J. J., Bao, W. J., Wang, F. B. & Xia, X. H. Catalyst-free synthesis of nitrogen-doped graphene via thermal annealing graphite oxide with melamine and its excellent electrocatalysis. *ACS Nano* **5**, 4350-4358 (2011).
10. Jiao, W., Li, N., Wang, L., Wen, L., Li, F., Liu, G. & Cheng, H. M. High-rate lithium storage of anatase TiO_2 crystals doped with both nitrogen and sulfur. *Chem. Commun.* **49**, 3461-3463 (2013).
11. Li, H., Zhang, X., Huo, Y. & Zhu, J. Supercritical preparation of a highly active S-doped TiO_2 photocatalyst for methylene blue mineralization. *Environ. Sci. Technol.* **41**, 4410-4414 (2007).
12. Tamor, M. A. & Vassell, W. C. Raman fingerprinting of amorphous-carbon films. *J. Appl. Phys.* **76**, 3823-3830 (1994).
13. Pimenta, M. A., Dresselhaus, G., Dresselhaus, M. S., Cancado, L. G., Jorio, A. & Saito, R. Studying disorder in graphite-based systems by raman spectroscopy. *Phys. Chem. Chem. Phys.* **9**, 1276-1291 (2007).
14. Mao, Y., Kanungo, M., Hemraj-Benny, T. & Wong, S. S. Synthesis and growth mechanism of titanate and titania one-dimensional nanostructures self-assembled into hollow micrometer-scale spherical aggregates. *J. Phys. Chem. B* **110**, 702-710 (2006).
15. Smirniotis, P. G., Sreekanth, P. M., Pena, D. A. & Jenkins, R. G. Manganese oxide catalysts supported on TiO_2, $Al2O_3$, and SiO_2: a comparison for low-temperature SCR of NO with NH_3. *Ind. Eng. Chem. Res.* **45**, 6436-6443 (2006).
16. Zhang, H., Ji, T., Liu, Y. & Cai, J. Preparation and characterization of room temperature ferromagnetic Co-doped anatase TiO_2 nanobelts. *J. Phys. Chem. C* **112**, 8604-8608 (2008).
17. Chen, S., Zhang, S., Liu, W. & Zhao, W. Preparation and activity evaluation of p-n junction photocatalyst NiO/TiO_2. *J. Hazard. Mater.* **155**, 320-326 (2008).
18. Ali, Z., Cha, S. N., Sohn, J. I., Shakir, I., Yan, C., Kim, J. M. & Kang, D. J. Design and evaluation of novel Zn doped mesoporous TiO_2 based anode material for advanced lithium ion batteries. *J. Mater. Chem.* **22**, 17625-17629 (2012).
19. Xiao, F. X. Construction of highly ordered $ZnO-TiO_2$ nanotube arrays (ZnO/TNTs) heterostructure for photocatalytic application. *ACS Appl. Mater. Interfaces* **4**, 7055-7063 (2012).
